# Insights into the Design of Polyurethane Dressings Suitable for the Stages of Skin Wound-Healing: A Systematic Review

**DOI:** 10.3390/polym14152990

**Published:** 2022-07-24

**Authors:** Maria Morales-González, Luis Eduardo Díaz, Carlos Dominguez-Paz, Manuel F. Valero

**Affiliations:** 1 Doctoral Program in Engineering, Faculty of Engineering, Universidad de La Sabana, Chía 140013, Colombia; mariamorgon@unisabana.edu.co; 2 Energy, Materials and Environmental Group, GEMA, Faculty of Engineering, Universidad de La Sabana, Chía 140013, Colombia; carlos.dominguez2@unisabana.edu.co (C.D.-P.); manuelvv@unisabana.edu.co (M.F.V.); 3 Bioprospecting Research Group, GIBP, Faculty of Engineering, Universidad de La Sabana, Chía 140013, Colombia

**Keywords:** polyurethane, urethane, wound dressing, wound healing, wound stages

## Abstract

Dressings made with polyurethanes have been found to exhibit good and varied biological properties that make them good candidates for this application. However, as has been seen, the wound-healing process is complex, which includes four different stages. So far, the design and evaluation of polyurethane for wound dressing has focused on achieving good properties (mechanical, physicochemical, and biological), but each of them separates from the others or even directed at only one of the stages of skin wound-healing. Therefore, the aim of this systematic review is to explore the applications of polyurethanes in wound dressings and to determine whether could be designed to cover more than one stage of skin wound-healing. The PRISMA guidelines were followed. The current research in this field does not consider each stage separately, and the design of polyurethane dressings is focused on covering all the stages of wound healing with a single material but is necessary to replace polyurethanes in short periods of time. Additionally, little emphasis is placed on the hemostasis stage and further characterization of polyurethanes is still needed to correlate mechanical and physicochemical properties with biological properties at each stage of the wound-healing. Current research demonstrates an effort to characterize the materials physiochemically and mechanically, but in terms of their biological properties, most of the literature is based on the performance of histological tests of explants morphologically probing the compromised tissues, which give an indication of the potential use of polyurethanes in the generation of wound-healing dressings.

## 1. Introduction

The process of wound-healing consists of four (4) phases: hemostasis, inflammation, proliferation, and remodeling. Those phases are dynamic and sometimes can overlap and involve the interaction of different compounds (clotting factors, growth factors, cytokines, chemokines), extracellular matrix (collagen), and cells (platelets, leukocytes, neutrophils, endothelial, monocytes, lymphocytes, macrophages, fibroblast, keratinocytes), among others [1,2].

First, in the hemostasis phase, the coagulation cascade is activated, and the platelet aggregation occurs to prevent blood loss [3]. The inflammatory phase starts when the injury occurs and is characterized by the secretion of cytokines from the immune cells and the removal of necrotic tissue, infection control through phagocytosis, and free radical production by the macrophages and neutrophils [4]. For the proliferation phase, many processes occur, such as re-epithelialization and granulation tissue and extracellular matrix (ECM) formation [4], due to the migration and proliferation of fibroblasts and endothelial cells to the wound site [3]. Finally, in the remodeling phase, also known as the maturation phase, both wound contraction and collagen remodeling (degradation of type III collagen and formation of type I collagen) occurs [3], and the tensile strength of the tissue increases [4].

The healing of the skin occurs naturally; however, in some occasions it is altered by different conditions or illness (diabetes, obesity, etc.) that delay this process [5]. Therefore, wound treatment and management has been an issue worldwide, with a need for improvement. Wound dressings are different materials that cover wounds to protect it from damage and pathogen attack and promote its healing [6]. Currently, a wide range of materials have been proposed for the design of wounds dressing, from traditional ones, such as gauzes, to smart wound dressings made from different natural and synthetic polymers. However, there is still no ideal wound dressing that can fit and heal all wound types [7], nor that suits all the stages of the wound-healing.

It has been found that, specifically, dressings made with polyurethanes present good and varied biological properties that make them good candidates for this application. Polyurethanes are one of the most widely used polymers in the market, since they have excellent mechanical flexibility, biodegradability, and physicochemical properties, which make them suitable for a wide range of applications such as fibers, coatings, and foams, among others [8]. Their synthesis process is characterized by the polycondensation reaction, in which urethane (or carbamate) bonds are generated between polyols and isocyanates [9]. Additionally, its structure is composed of hard and soft segments that generate better mechanical properties. Therefore, its versatility is wide, as the quantities and ratios of the polyols and isocyanates used can be adjusted [8]. The biocompatibility and biostability of polyurethanes make them promising for application in the biomedical and clinical fields, which has led to recent increasing demand for them [9,10].

However, as have seen, the wound-healing process is complex, and each wound healing stage has specific molecular and physiological processes that demand different properties from dressings [6]. So far, the design and evaluation of biomaterials for wound dressings has focused on achieving good mechanical, physical, and biological properties, but each of them separately and without considering the requirements of each stage of wound-healing, so the dressings that have been developed from these efforts are not easily adaptable to different environments or stimuli [6], and they must be replaced in a short time.

Thus, the systematic review aims to explore:

- Is it possible that a single polyurethane dressing can help each stage of the wound-healing process?
- Should the polyurethane wound dressing design be based on each stage of the wound-healing process?
- What properties or characteristics should a polyurethane dressing have to perform well in each stage of the skin wound-healing process?

The purpose of the systematic review is to examine and explore the applications of polyurethanes in the stages of wound healing.

## 2. Methods

### 2.1. Search Strategy

A search of reports from 2012 to 2022 was conducted on Scopus, Web of Science, PubMed, and Sage Journals databases on 22 January 2022, according to PRISMA guidelines. For Scopus, Web of Science, and Sage Journals databases, the search equation used was ((polyurethane OR urethane) AND (dressings OR “wound dressings” OR “wound healing”)), and for PubMed, the search equation was (((((polyurethane OR urethane) AND (dressings OR “wound dressings” OR “wound healing”))) NOT (review [Publication Type])) NOT (Systematic Review [Publication Type])) NOT (Meta-Analysis [Publication Type]). The searches were limited to research articles or original articles. Case studies were considered if they met inclusion/exclusion criteria.

### 2.2. Inclusion and Exclusion Criteria

Inclusion criteria were as follows: (1) research with information on polyurethane synthesis and characterization; (2) evaluation of biological activities such as inflammation, hemocompatibility, tissue granulation, and/or tissue remodeling; (3) in vitro and in vivo biological assays.

Exclusion criteria were as follows: (1) reports wrote in other languages besides English; (2) short reports; (3) reviews, systematic reviews, and/or meta-analysis; (4) in silico assays; (5) the biological activities are of the polyurethane and not of another compound and are not simply a support for other compounds or materials that have those properties.

### 2.3. Selection and Data Collection Process

The selection of the reports was divided in two phases. The first phase was a blind title and abstract review by three researchers independently and in accordance with the inclusion and exclusion criteria. The discrepancies between review authors were solved through discussion.

In the second phase, the potentially eligible reports were full text examined by one researcher for the final selection of reports to be included. For the data extraction of the included reports, a data extraction form was created and reviewed by the three researchers. The data were tabulated using the form by one researcher and verified by the other two. The review was not registered.

## 3. Results

### 3.1. Selection and General Characteristics of the Included Reports

**Figure 1:**
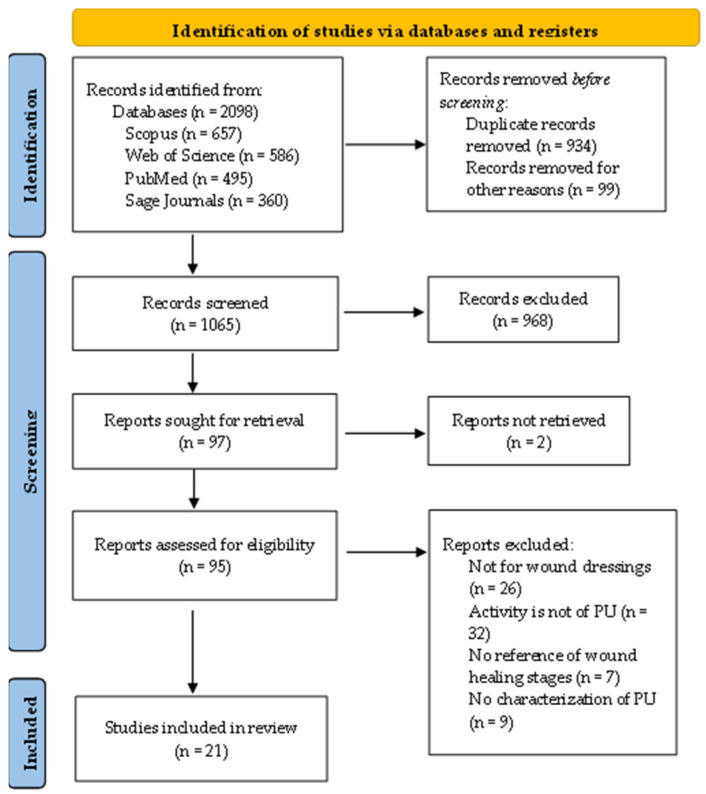
Flow diagram of the systematic literature search according to PRISMA guidelines.

After manual retrieval, initial database search identified 2065 relevant reports of which a total of 1065 non-duplicate and English reports were screened (Figure 1). Of those, 968 were excluded and 95 were included for further examination for eligibility. Finally, 21 reports were selected based on the inclusion/exclusion criteria and were included for data extraction.

Regarding the reports excluded upon full-text examination, as shown in Figure 1, the reasons include polyurethanes not being for wound dressings applications (*n* = 26), lack of interest in the stages of wound-healing (*n* = 7), lack of mechanical, physicochemical, or thermal characterization of the polyurethane (*n* = 9) and finally, the biological activities that were evaluated not being from the material but from some other compound or added material (*n* = 32).

**Figure 2:**
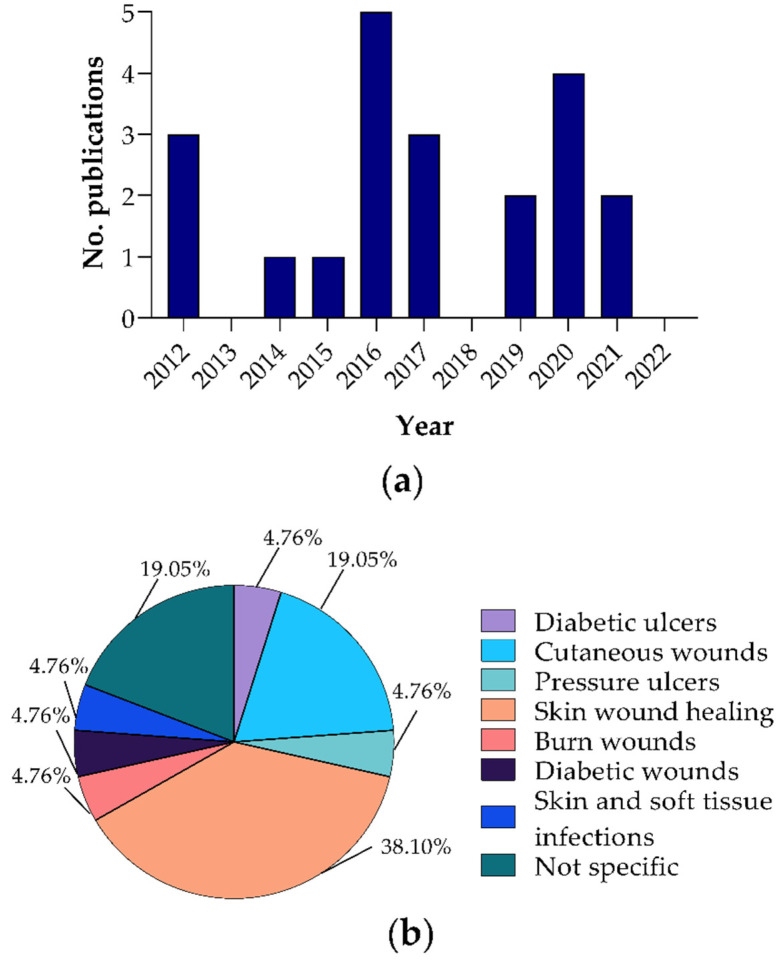
Distribution of reports of polyurethane for wound dressings application (**a**) in this systematic review per year; (**b**) according to the type of wound.

According to the time frame selected for the systematic review, the included reports were published between 2012 and 2021 (Figure 2a). Interestingly, in the years 2013 and 2018, there were no publications regarding polyurethanes with biological activity in the wound dressing that were characterized mechanically and/or physiochemically; an oscillating behavior of the publications on this subject can be observed. As seen in Figure 2a, the most reports (*n* = 5) were published in 2016, followed by 2020 (*n* = 4), decreasing in 2021 (*n* = 2). Likewise, most of the research was done in China (*n* = 8), followed by Iran (*n* = 7), USA (*n* = 3), and India, Canada, United Kingdom, and Germany with one report each. The main type of wound evaluated in the reports are the skin wounds, as shown in Figure 2b, with 38.10% corresponding to eight reports. Four reports evaluated cutaneous wounds (19.05%) and four reports did not specify the type of wound. The other evaluated wounds were diabetic ulcers, pressure ulcers, burn wounds, diabetic wounds, and skin and soft tissue infections (one report, 4.76%).

### 3.2. Polyurethane Dressings

Different types of polyurethanes have been evaluated for the generation of wound dressings. Table 1 shows the summary of the type of polyurethanes studied, the modifications made, and the synthesis techniques used. For the preparation of the dressings, commercial polyurethane has been used to a greater extent (*n* = 11), and in those that involve the synthesis of the polyurethane, the most-used isocyanate is 1,6-hexamethylene diisocyanate (HDI) (*n* = 5), followed by L-lysine-based diisocyanates (LTI) (*n* = 3). Two of the studies made polyurethanes from vegetable oil monomers such as castor oil and soybean oil. Likewise, the most-used polyols for the manufacture of polyurethanes are polyethylene glycol (PEG) and polycaprolactone (PCL).

Considering the monomers used and the types of wounds selected, the types of dressings generated with polyurethanes focus on films (*n* = 5), scaffolds (*n* = 5), foams (*n* = 5), nanofibers (*n* = 3), porous membranes (*n* = 1), waterborne dressings (*n* = 1), and injectable scaffolds (*n* = 1). For these, synthesis processes such as electrospinning for nanofibers, particle leaching and reactive liquid molding for scaffolds, porous membranes and injectable scaffolds, and two-step polymerization for films are used (Table 1). For example, isocyanates (HDI or LTI) are used for the synthesis of films and some scaffolds, but commercial polyurethanes were used for the synthesis of the nanofibers. L-Lysine isocyanate was used for the injectable scaffolds and the waterborne dressings.

Regarding the mechanical properties evaluated in wound dressings from polyurethanes, the tensile strength was in a range between 1.7 MPa and 34 MPa (Table 2). In the case of the scaffold generated by Bankoti et al. 2017, it can be observed that between the dry and wet polyurethane there is a great difference in the mechanical properties, once the polyurethane is wet, the tensile strength decreases considerably between 70.5 and 73.1%, but the elongation at break increases between 79.0 and 80.0% [12]. Foams, porous membrane, and scaffolds, which are porous matrices, did not show tensile strength greater than 8 MPa, while waterborne dressings and films show a wide range of tensile strength (1.7 to 17.8 MPa).

Likewise, in terms of water absorption and contact angle, it can be seen that polyurethanes have high water absorption with values ranging from 0.5% to almost 700%. Those polyurethanes synthesized from PEG present high values of water absorption, and together with this, the values of the contact angle also vary, having values for these polyurethanes in the range of 41.4° to 98.3°. As for the other synthesized polyurethanes without any type of modification, the values of the contact angle are in a wide range, with maximum values of 98° to 118°. The water vapor transmission rate, which has lately been found to be one of the most important properties in the design of materials for wound dressings, can be observed in Table 2 to be in the range between 50 and 900 g/m^2^ day.

Additionally, within the porous polyurethanes, scaffolds, nanofibers, and foams, the pore size ranged from 1.41 μm to 423 μm. In most cases, spherical, and interconnected pores were observed. Some had structures that are like cell structures, and some had an open-cell structure.

For most of the cases, some type of modification was made to the polyurethane. Mainly, there was the incorporation of some compound or extract that provides antibacterial properties to the polyurethane. Although one of the exclusion criteria referred to the fact that the biological activity should be due to the polyurethane, in the articles included, the addition of this compound/extract produces the antibacterial properties and has little influence on the wound-healing properties, without affecting their mechanical properties.

Therefore, ethanolic and water extract of propolis, *Malva sylvestris* ethanolic L. extract, chitosan, mupirocin, quaternary ammonium salts, and bromelain–ferula gum were used to improve the antibacterial activity against *E. coli* and *S. aureus*. *Malva sylvestris* L. ethanolic extract was incorporated at concentrations of 5, 10, 15, and 20% *w*/*w* [11], chitosan in a ratio ranging from 0 to 10, and mupirocin solutions at 0, 2, and 5% (*w*/*w*) [12]. For the use of propolis extracts, one of the reports evaluated water extracts at 0%, 10%, 20%, and 30% [17], and the ethanolic extract was made at a 1:10 ratio (25 g of propolis in 250 mL of ethanol) [15] and at 0.5, 1, and 2% *w*/*w* [20]. The results of these studies show that polyurethanes do not possess antibacterial activity on their own, which is important for the design of wound dressings. For example, with the use of 20% of *Malva sylvestris* L. extract, the activity against *E. coli* and *S. aureus* could improve to nearly 70% [11].

**Figure 3:**
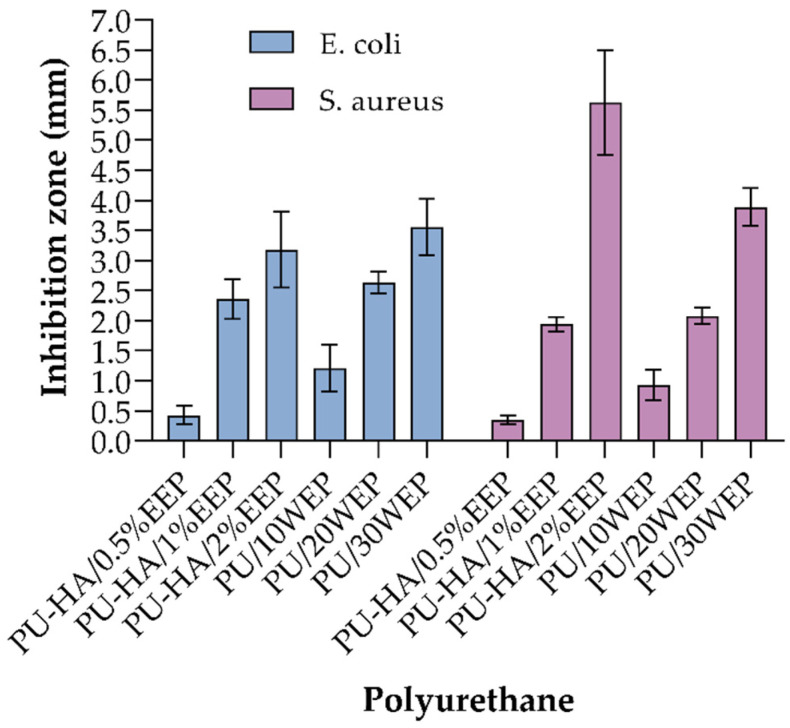
Comparison of antibacterial activity against *E. coli* and *S. aureus* of polyurethanes with incorporation of ethanol and water propolis extract. EEP refers to ethanolic propolis extract and WEP refers to water propolis extract. Figure constructed by the authors based on data from the articles [17,20].

Comparing the results of antibacterial activity of polyurethanes with incorporation of propolis extracts, it can be observed, as shown in Figure 3, that against *E. coli*, the activity is similar for ethanol and water extracts, even with the differences in concentration. For all cases, the activity increases with increasing concentration of extract in the polyurethane. However, the polyurethanes with 2% propolis ethanol extract (3.18 ± 0.63 mm) and 30% propolis water extract (3.55 ± 0.47 mm) achieved similar activity against *E. coli*. As for the activity against *S. aureus*, polyurethanes with 2% propolis ethanol extract reached an activity of 5.63 ± 0.87 mm at the highest, followed by polyurethane with 30% propolis water extract 3.89 ± 0.31 mm. Pahlevanneshan et al. obtained similar results; they made polyurethanes with ethanolic propolis extract and compared them with the inclusion of water propolis extracts, reporting that higher activity was obtained with ethanolic extracts than with water extracts [15].

Another modification that is made to polyurethanes for dressings are surface modifications. In this case, plasma pre-treatments and implementation of cationic, anionic, and zwitterionic surfaces were used. These modifications allow different interactions of the polyurethane with different host cells and bacteria, which make available, in many cases, improvements to biological activities.

### 3.3. Polyurethane Dressing in Wound Healing Stages

Regarding the biocompatibility assays performed in the reports, cell viability was measured on them with the MTT (3-[4,5-dimethylthiazol-2-yl]-2,5 diphenyl tetrazolium bromide)assay, CCK-8 assay, and LIVE/DEAD staining assay, against the cell line’s mesenchymal stem cells from the human umbilical cord matrix (hUCM), fibroblasts from mouse (L929 cell line, NIH 3T3 and directly isolated from Balb/c neonatal mice), human skin keratinocytes (HaCat), Human Umbilical Vein Endothelial Cells (HUVECs), macrophages, endothelial cells, and fluorescent human embryonic kidney (HEK293) cells.

Of the reports reviewed, 13 performed a cell viability or proliferation assay; of them, all the synthesized polyurethanes presented high biocompatibility, with high cell viability and cell proliferation without any toxic effects against the cell lines studied. For example, in the L929 cell line, the cell viability was in the range of 68.64% to almost 200%. The polyurethane that presented lower cell viability (68.64%) at day 1 was porous polyurethane-urea foam (PUUF) with NIH 3T3 cells; however, after 3 days, a cell viability of 80.57% was achieved.

Proliferation values were in a range of 88.8% to 900%, depending on the cell line. In the case of the human umbilical cord matrix (hUCM) after 7 days of culture, the increase of the cells in the polyurethane was of 88.8%. Fibroblasts were isolated from normal foreskin showed proliferation of 546.6%, while for L929, a 900% increase in the proliferation was reported.

**Figure 4:**
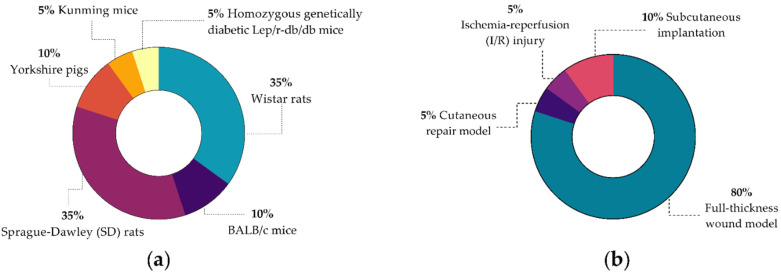
(**a**) Reports by the animal model used in the in vivo evaluation of polyurethane for wound dressing applications and (**b**) Reports by injury model applied in the in vivo test for the evaluation of polyurethanes as wound dressings.

For the biological evaluation of polyurethanes as wound dressings, different animal models have been used (Figure 4a), mainly murine models such as Sprague–Dawley rats, Wistar rats, and BALBB/c mice. Two reports performed in vivo evaluations in a pig model with Yorkshire pigs. Also, as can be seen in Figure 4b, the most-used wound model for in vivo evaluation of polyurethanes in wound-healing applications is the full thickness wound model (*n* = 16). This is followed by a subcutaneous implantation of the materials. Only one report performed an ischemia–reperfusion injury model.

An important aspect of wound treatment with wound dressings is the change that should be made to the dressing. For the evaluations made in the included reports, 10 of them do not specify if any change was made or not, so it can be considered that the same dressing was used during the entire treatment time. Those who mentioned dressing changes performed them every day (*n* = 1), every other day (*n* = 1), every two days (*n* = 3), every 2, 3, or 4 days (*n* = 3), or every 7 days (*n* = 1). However, the schedule for dressing changes is also related to the treatment time. Normally, the healing process of a wound, from the beginning of the wound to its closure and if no adverse event occurs, has a duration of 21 days. For the synthesized polyurethanes and the models used, the treatment time differed, from 3 days to 30 days (Table 3).

**Figure 5:**
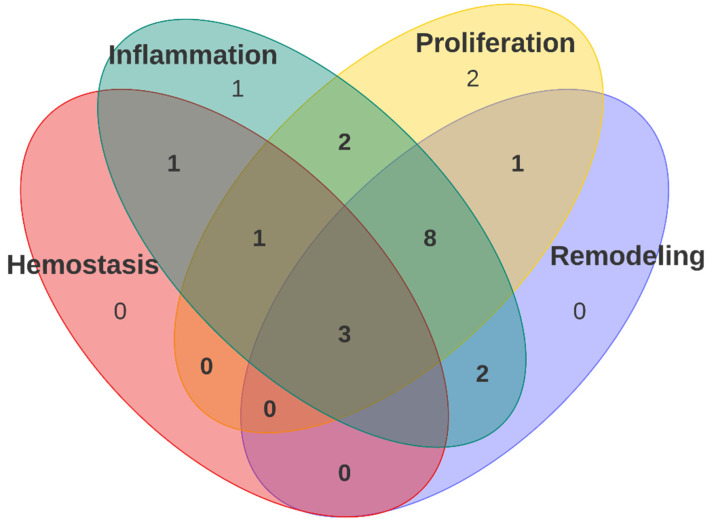
Number of reports that performed any biological evaluation of polyurethanes for each stage, two stages, three stages, and all four stages of the wound-healing process.

Beyond treatment for the biological evaluations, the tests performed on animals and tissues are also similar in all reports. Most of them perform histological analysis of the tissue after the treatment time with different staining, i.e., hematoxylin and eosin (H& E) for cell nuclei, extracellular matrix, and cytoplasm, Toluidine Blue for epithelialization, and Masson Trichrome for collagen fibers. Very few perform any other type of analysis such as cytokine release or gene expressions. Therefore, the evaluation of the polyurethane is not performed by wound-healing stages. Although histological analysis allows the evaluation of different aspects of wound-healing, a complete characterization of each stage of the wound-healing is not performed. As shown in Figure 5, of the total number of reports included, only five reports evaluate the first stage of the healing process, which is hemostasis. This evaluation is generally in vitro, with hemocompatibility assays such as clot formation, platelet adhesion, and hemolysis. Next, the inflammation and proliferation stages are the most evaluated, with a total of 18 and 17 reports, respectively, and finally, the evaluation of the remodeling process was presented in 14 of the included reports.

Also, of the 21 included reports, just 3 mention any evaluation for the four stages of the wound-healing process; however, the evaluation performed was more qualitative than quantitative, and none of them allow for correlation of the polyurethane properties (mechanical and physicochemical) with the biological behavior, which would enable the establishment of metrics for the evaluation and comparison of polyurethanes for wound dressing applications in the design and generation of the ideal polyurethane wound dressing. Of those three reports, only Chen et al., 2017, presented a different test from histological analysis to evaluate any quality of the four stages. They evaluated the expression of proinflammatory cytokines TNF-α and IL-6 after 3 days, even thougth the histological analysis revealed that the polyurethanes did not showed an inflammatory reaction, and the study of these cytokines revealed that the incorporation of mupirocin in the matrix will induce infection and it could delay the wound-healing process, in comparison with the polyurethane alone.

For the case of the reports (*n* = 8) that mentioned three stages (Figure 5), all of them conducted a histological analysis that mentions the observation of low inflammatory reaction, granulation tissue formation, epithelialization, angiogenesis, and collagen deposition.

Within the evaluation of the inflammation stage, there are analyses that include the inflammatory reaction, with the observation of different inflammatory cells in the tissue, the release of pro-inflammatory and anti-inflammatory cytokines such as TNF-α, IL-10, and IL-6, and/or the cellular infiltration in the tissue of cells such as keratinocytes, fibroblasts, and macrophages. Some of the studies evaluated the behavior of macrophages, i.e., observed their phenotype change and whether the change to an inflammatory phenotype was promoted by polyurethane [18]. The results showed a higher cellular infiltration, a lower number of inflammatory cells, and a lower immune reaction of the tissue with the different polyurethanes.

For the evaluation of the proliferation stage, mainly the observations of epithelialization and neovascularization were presented. The formation of epithelium in the wound area and the formation of new blood vessels in this space were verified by different stains. Neovascularization and epithelialization were more pronounced, and, in some cases, increased fibroblast infiltration, adhesion, and proliferation were seen. Finally, for remodeling, the collagen deposition in the wound area (which in most cases was investigated via Masson Trichrome staining) and the formation of granulation tissue and its thickness were considered, where a greater formation of granulation tissue and tissue repair was observed. Likewise, collagen deposition was higher in wounds treated with the synthesized polyurethanes.

The most-used test for in vivo evaluation of wound dressings is the monitoring of wound closure (area of closure). Fifteen reports performed this evaluation, and as can be seen in Table 3, the wound closure values for polyurethanes are above 70% (Table 3). Likewise, in most cases, the control used in the in vivo test is gauze, and low wound closure results are observed (below 70%). Some of the polyurethanes, such as polyurethane/carboxymethyl cellulose (PU/CMC) composite nanofiber with malva extract, waterborne polyurethane-chitosan hydrogel scaffolds, high porous polyurethane, electrospun polyurethane–hyaluronic (PU–HA) acid nanofiber, biodegradable electroactive polyurethane-urea elastomers, castor oil PU membranes films, and porous polyurethane–urea foam (PUUF), have wound closure percentages above 90%. These good results from the use of diverse types of polyurethanes for wound closure demonstrate their potential as wound dressings for various wounds, such as skin wounds, diabetic ulcers, pressure ulcers, burn wounds, diabetic wounds, and skin and soft tissue infections.

## 4. Discussion

The synthesis of polyurethanes is based on the polycondensation reaction, by which polyols react with isocyanates to generate the urethane bond [8]. According to these monomers, the resulting properties will vary, making them attractive to industry in a wide range of applications. Currently, the search for better monomers that give better properties is one of the concerns in the field of polyurethanes, and on them depend on the biological applications that can be had. Based on the global distribution of the included publications, it is notable that no investigations regarding polyurethane for wound dressing applications have been made in Latin American countries. The development is centered in Asia, followed by North America. Thus, there is an opportunity in Latin American countries to generate investigation in this field.

In the case of the isocyanates used, HDI is an aliphatic isocyanate that is widely used because of its nontoxic biodegradation product, 1,6-hexanediamine [32]. The polyurethanes synthetized have well-ordered hard segments by hydrogen bonds that generate high elasticity and strength due to their symmetrical chemical structure [32], giving them desirable mechanical properties in biomedical applications that, in many cases, are not achieved with other types of materials. In the same way, the use of L-lysine-based diisocyanates has demonstrated advantages in terms of environmental aspects, as it is a greener option for obtaining isocyanates, and it has been found that it can give polyurethanes similar properties to those of polyurethanes made with HDI or isophorone diisocyanate [33]. Also, the use of lysine diisocyanate has resulted in the generation of biodegradable and biocompatible polyurethanes, which enable them to be used in biomedical applications [23,34,35].

Polyols such as polyethylene glycol (PEG) and polycaprolactone (PCL) in biomaterials have been in demand due to the high biological properties they confer to polyurethane. PEG is a polyether polyol that can produce polyurethanes characterized by low-temperature flexibility, high hydrolytic stability, and resistance to fungi and bacteria attack [36], this last one being among the most important activities sought after in wound dressings. Another important aspect of PEG is its ability to hydrate generating structures that physically and chemically resemble the extracellular matrix (ECM), which is important in the design of biomaterials [37]. In contrast, PCL is a polyester polyol that provides high strength and biodegradation to the polyurethane synthetized. Currently, is widely used for biomedical applications owing to its high biocompatibility [38,39]. The mixture of PEG and PCL in the polyols used for the synthesis of polyurethanes has shown good results in terms of mechanical and biological properties. For example, the use of PEG in polyurethane increased the swelling of the material [40,41], which is a benefit for wound dressings because it allows for exudate and fluid exudate absorption.

However, none of the polyurethanes synthesized for wound dressing applications are from renewable sources. One of the problems currently facing these materials is the growing concern over the use of monomers from petrochemical sources and the use of isocyanates that have been shown to be carcinogenic [42]. Thus, one of the greatest challenges in the field of biomaterials as wound dressings is the search for new synthesis routes of polyurethanes with renewable monomers and avoiding the use of isocyanates, but that allow preserving the good mechanical, physicochemical, and above all, biological properties that polyurethanes provide in wound-healing.

Regarding the mechanical properties of polyurethanes, it can be observed that the tensile strength of films, scaffolds, and foams show values comparable to the values reported for normal human skin, between 2.5–16 MPa [31]. Although there are no major differences between the type of dressing generated from polyurethanes, the nanofibers showed higher tensile strength, between 5 to 27 MPa. Thus, mechanically, wound dressings generated from polyurethanes are applicable on human skin. Likewise, for water vapor transmission rates for polyurethane films, as with the other properties, the values differ greatly. In the case of nanofibers made from polyurethane/carboxymethylcellulose with the addition of malva extract, the values are too high (between 1600 and 2000 g/m^2^ day), which differs greatly from the value of normal human skin, which is in the range of 204 g/m^2^ day [31]. On the other hand, the films made from castor oil and CAPA oil present a value very similar to this (260 and 285 g/m^2^ day, respectively).

A notable aspect of the investigation in the field of polyurethanes for wound dressing applications is the increasing interest in the modification of the matrix with different compounds or materials to improve the different biological activities required. In wound dressings, bacterial infection plays an important role in the healing process. When there is a bacterial infection in a wound, it mainly prolongs the healing process. This is due to the formation of the organized structure of bacterial biofilms that adhere to the tissue and biomaterial and prevent the effect of antibiotics [43]. Typically, the antibiotics used act on bacteria by disrupting the function of the bacteria’s structure, such as walls, or their metabolic pathways [44]. The most common pathogens encountered in bacterial wound infection problems are *Staphylococcus aureus* and *Escherichia coli*, so the antibacterial evaluation of wound dressings generated has focused on these two [15].

In the case of *S. aureus*, it has been seen that it is through biofilms that colonization of the wounded skin begins, mainly influenced by the hydrophobic and hydrophilic interactions of the tissue [45]. Thus, one of the mechanisms that can be studied in polyurethanes to improve their antibacterial activity against this pathogen is the hydrophobic and hydrophilic characteristic of the surface. As for plant extracts generated from water or ethanol, it has been seen that the improvement in bacterial activity by ethanol extracts may be due to the presence of phenolic compounds or flavonoids [15]. The difference between the activity against *S. aureus* and *E. coli* is mainly due to the structure of the wall. *S. aureus* has a single peptidoglycan layer, while the *E. coli* wall is composed of a thick layer of lipopolysaccharides, which makes it more resistant to hydrophobic compounds (present in ethanol extracts) compared to *E. coli* [15]. However, the extraction of highly polar compounds with water can generate extracts with greater antibacterial activity against *E. coli* in comparison with *S. aureus*. Thus, due to the permeability of the walls, Gram-negative bacteria are usually less susceptible to antibacterial agents than Gram-positive bacteria [46].

Nevertheless, it has been seen that the incorporation of some antibacterial compounds by methods such as leaching are not a viable option over the long term because, with the release of the compound over time, the material loses antibacterial activity, and eventually, the bacteria may become resistant to this compound when it is in low concentrations; also, some of them can become toxic to other cells present in the wound [32]. Therefore, it would be desirable to design materials that present antibacterial activity without the dependence on compounds–extracts foreign to the matrix, which would allow for overcoming these events.

As seen in this review, many of the polyurethanes can have good biological activities, as well as biocompatibility with different cell lines. However, these evaluations have focused on murine cell lines, such as murine fibroblasts, macrophages, and/or endothelial cells. Therefore, for the next step in the design of wound dressings that can reach a human application and that allow for the understanding of the dressing–host environment relationship, it is necessary to implement more evaluations in human cell lines such as HaCat, HDFa, HUVEC, among others. Xu et al., Hao et al., and Almasian et al. presented good results in these lines, with no toxic effects and with good adhesion and even proliferation of the cells, which is desirable in these applications.

Thus, for the implementation of any material in medical care, its biological activity and its correct evaluation is a crucial step. For the case of wound dressings and wound-healing systems, different models have been postulated in which different animal models have been used. As mentioned above, the use of murine models is highly accepted due to their ease of access. Some of the features of murine models include cost-effectiveness, ease of animal maintenance and handling, extensive literature for the procedure, and a relatively simple surgical process [47,48]. However, the major disadvantage of using these models is the thickness of the skin of the animals and the presence of the dense layer of hair. Compared to human skin, the skin of rodents or rats is only 10–15% as thick, which limits the size of the wound and the wound model to be performed [47]. Therefore, the full thickness wound model must be performed and it is not possible to perform partial thickness models, as observed in the models used for the evaluation of polyurethanes, where 80% corresponds to the full thickness model. The full-thickness wound model consists of complete removal of the epidermis and dermis to the depth of fascial planes or subcutaneous fat and disruption of dermal blood vessels. Because of this, this model has been implemented in most wound-dressing studies as it allows macroscopic measurement of the effects of treatment with the excision area, as well as monitoring of granulation tissue formation and re-epithelialization [49]. It allows easy collection of tissue for histological analysis to observe angiogenesis, collagen, and connective tissue content. Therefore, large, and deep wounds can be made that include all stages of the wound-healing process [49]. Likewise, the location of the wound is limited by having a much smaller size; in this case, rats offer greater area for wounding, allowing the generation of multiple wounds in a single animal [47,50].

The major concern with these types of models lies in the difference between the physiological mechanism of healing between rodents/rats and humans, mainly in terms of healing times [47]. In these animals, the process will always be much more accelerated than in humans, so estimating the treatment time of a biomaterial can never be done in these models. This can be seen in the different in vivo trials that were performed in the reports included in this review, where at short times, a significant reduction in the wound area performed was already observed. Although this can be attributed to the effect of polyurethane, it is not possible to establish this by histological analysis alone.

Therefore, the use of different animal models with a closer resemblance to human biology and the human immune response has been evaluated. Here, the use of porcine models offers a way to overcome the limitations of the murine model, such as the size of the animal, that allows for multiple interventions in a single animal, which decreases the number of animals required [50]. However, this presents disadvantages as well, such as the high cost in breeding and maintenance of animals, biosafety issues being more difficult to ensure, and the availability of animals with different ages and reagents for further analysis [50]. However, as can be seen in the reports by Adolph et al. 2014 and Adolph et al. 2016, the use of this model may be appropriate for the evaluation of polyurethanes as wound dressings.

None of the included reports used specific assays to evaluate the different characteristics of the four stages of the wound-healing process. Although three of the reports mentioned results from all four stages, the results were qualitative, many of them with histological analysis, and there is no possibility to compare the results between polyurethanes. Also, they lack a full characterization of the behavior of the polyurethane in the specific molecular and physiological processes of each stage. Some of them generated specific assays to quantify angiogenesis and neovascularization; however, as mentioned above, they do not perform assays for hemostasis.

Despite this, these reports provide a good approximation for the design of polyurethanes for wound dressings and allow postulating polyurethanes as ideal candidates for use in this application. They provide a starting point to contemplate various aspects in the design and synthesis of polyurethane that allow its complete characterization in terms of its behavior against each stimulus to which it could be subjected at each stage of the healing process.

Finally, changing dressings at the time of treatment is one of the aspects that most affect the effectiveness of a wound dressing. Ideally, a dressing should be able to act for the entire time of healing without the need for a change. According to the results observed in the reports included in the review (Table 3), it is conceivable that wound dressings made of polyurethanes could be changed according to the stage in the wound healing process, so polyurethanes could be designed considering the requirements of each stage.

Based on the results obtained regarding the porosity of polyurethanes, as well as their contact angles, water absorption, and WVTR, polyurethanes could cover different stages of the wound-healing process. For the hemostasis stage, it has been observed that the surface of polyurethanes helps in the absorption of proteins, especially in the absorption of fibrinogen, which triggers platelet adhesion in the wound. Likewise, platelet activation facilitates thrombin formation, platelet aggregation, and blood clotting [24]. Therefore, polyurethanes with good water absorption facilitate this stage. Those materials with water absorption higher than 100% and with a fast absorption rate (less than one day) allow an adequate course of the hemostasis process and favor wound-healing, since it has been seen that this generates the ability to absorb the water present in the blood and thus favor the concentration of the precursor compounds that activate the coagulation cascade at the site of bleeding [24], allowing shorter bleeding times.

As for the inflammation and proliferation stage, the pore size plays a fundamental role for cellular infiltration and thus the adaptation of the foreign body in the human body. It has also been shown that pore size is related to wound moisture, high protein adsorption, oxygen penetration, and the exchange of nutrients and waste at the wound site [12,13,14,17,20]. Therefore, polyurethanes with open-cell and interconnected pore structures increase cell growth, water absorption capacity, and high moisture vapor transmission rate, which allow for high absorption of exudates in wounds and thus create a moist environment that prevents drying and scar formation in the wound [15]. Similarly, this moistness being absorbed by polyurethane helps to prevent infection and the proliferation of microorganisms in the wound by the low water activity at the wound site [15].

To this end, it has been seen that those polyurethanes with pore sizes approximate to 5 μm can induce greater neovascularization, which in turn generates greater tissue remodeling and the formation of a new dermis like the original tissue [12]. Additionally, the neovascularization can be obtained with polyurethanes with pore sizes between 50 and 350 µm [15].

One of the fundamental advantages of polyurethanes and the reason why they have been catalogued as highly biocompatible materials is the non-toxicity of these materials, which helps in avoiding a high and prolonged inflammatory response [23]. Likewise, the final structure of polyurethanes resembles the human extracellular matrix and allows a mimicry of the natural structure of the skin that helps the wound-healing process to be faster and better [13,15,18].

Therefore, for the inflammation and proliferation stages, polyurethanes with hydrophilic surfaces help establish direct contact with the wound area and therefore a better adaptation to the body [15], providing a moist environment that enhances adhesion, migration, and proliferation of the different cells involved in these stages such as fibroblasts and keratinocytes, allowing the regulation of different genes such as E-cadherin, proliferating cell nuclear antigen (PCNA), and production of epithelial growth factor (EGF) that help in improving the wound-healing process [15,16]. Regarding the swelling of polyurethanes, those with high swelling rates allow high absorption of exudates, which promotes tissue regeneration [21]. This suggests that for these stages, it is desirable to have polyurethanes with high swelling rates (greater than 100%).

Polyurethanes with contact angles between 40–80° with high water absorptions and WVTR between 300 g/m^2^ day [22] would be candidates to enhance the stages of inflammation, proliferation, and remodeling. Likewise, the similarity of polyurethanes with the mechanical properties of the skin allows for better organization of fibroblasts in the wound, generating an adequate deposition of the extracellular matrix [25], which has a positive impact on scar formation and re-epithelialization of the wound.

However, detailed mechanisms of the action of polyurethanes in these wound-healing processes have not yet been developed. There is still a need to generate mechanisms that allow for the understanding of the relation between WVTR and the hydrophilicity of polyurethanes in the molecular pathways for the activation of the different type cells that improve the adhesion, proliferation, and migration of these cells. It is important to highlight that the most impactful of these dressings, as mentioned above, consist of polyurethanes modified with bioactive compounds, since the polymer by itself does not show activity.

## 5. Conclusions

The current research in this field does not consider each stage separately and the design of polyurethane dressings is focused on covering all the stages of wound-healing with a single material, but it is necessary to replace polyurethanes in short periods of time. Additionally, little emphasis is placed on the hemostasis stage. For that reason, some important aspects can be considered for the design of polyurethanes for wounds dressings as follows: (1) the selection of monomers that provide good mechanical properties, similar to those of human skin, physicochemical properties that allow an adequate similarity with the host environment and that allow an adequate exchange of gases and fluids; (2) the investigation of new synthesis routs that allow the generation of polyurethanes without petrochemical source or without the use of harmful isocyanates; (3) the search for strategies to improve the antibacterial activity without the inclusion of any extract or compound modifying the other properties; (4) characterization the performance of the materials in each stage of the wound-healing process in order to generate polyurethanes that do not need replacement during the treatment; (5) generation of techniques that allow for the evaluation of polyurethanes in each stage of the healing process; (6) the opportunity for countries in the Americas to generate research in this field, which could generate impacts on the health of their population; (7) further research on the mechanisms of polyurethanes at each stage of the wound-healing process, with assays and results that allow the relationship of polyurethane characteristics to the observed biological response, detailing the processes that are favored at each stage of wound-healing. This review highlights the great potential that polyurethanes have to serve as wound-healing dressings by considering each of the stages of this process compared to other types of polymers, since their synthesis can be directed with respect to the desired biological, mechanical, and physicochemical characteristics. The mechanism for wound-healing of polyurethanes is given by the favoring of cell adhesion and proliferation processes, both of fibroblasts, keratinocytes, and monocytes. Thus, to accelerate the hemostasis process, it is preferable to use polyurethanes with high water absorption rates to activate the coagulation cascade and stop bleeding in less time. Polyurethanes with contact angles between 40–80°, high water absorption, and WVTR (300 g/m^2^ day) would be candidates to improve the stages of inflammation, proliferation, and remodeling.

## Figures and Tables

**Table 1:** A summary of studies of polyurethanes for wound dressing applications with evaluations of the stages of wound-healing.

Authors	Polyurethane	Monomers	Synthesis Technique	Modification	Wound Type	Dressing Type	Study Design	Wound Area	Reference
Almasian et al., 2020	Polyurethane/carboxymethyl cellulose (PU/CMC) composite	Polyurethane (MW = 110,000) and carboxymethyl cellulose (CMC)	Electrospinning	*Malva sylvestris* extract	Diabetic ulcers	Nanofibers	In vitro In vivo	Circle: 1.5 cm diameter	[11]
Bankoti et al., 2017	Waterborne polyurethane-chitosan hydrogel scaffolds	Chitosan (MW 7,10,000) and polyurethane diol aqueous dispersion	Mechanical blending and casting	Chitosan	Skin wound healing	Scaffolds	In vitro In vivo	Square: 2 cm^2^	[12]
Lei et al., 2016	Biomimetic porous membrane composed of thermoplastic polyurethane (TPU)	Thermoplastic polyurethane granules (TPU)	Immersion precipitation and particle leaching	Na–citrate powder	Cutaneous wounds	Porous membrane	In vitro In vivo	Circle: 0.4 cm diameter	[13]
Mousavi et al., 2021	PU-Br-Fg electrospun nanofibers	Biocompatible PU resin (Desmopan 9370A) and Polyvinyl alcohol (PVA 67000)	Electrospinning	Bromelain (Br) and Ferula gum (Fg)	Pressure ulcers	Nanofibers	In vitro In vivo	N/A	[14]
Pahlevanneshan et al., 2021	Nanocomposite PU foam	Polyethylene glycol (PEG 400 and PEG 600), glycerol, 1,6-hexamethylene diisocyanate (HDI)	One shot and solvent-free foam preparation and PU foams coating and soaking	Nanolignin; coated with ethanolic extract of propolis	Skin wound healing	Foam	In vitro In vivo	Circle: 1.1 cm diameter	[15]
Xu et al., 2016	Microporous PU membranes	Medical-grade PU	Particulate leaching method	Sodium citrate	Skin damage	Film	In vitro In vivo	Square: 1 cm^2^	[16]
Khodabakhshi et al., 2019	Highly porous polyurethane	Polyurethane (Tecoflex EG-80 A)	Solvent casting/particulate leaching	Coated with the water extract of propolis	Skin wounds	Foam	In vitro In vivo	Circle: 1.1 cm diameter	[17]
Guo et al., 2015	Poly(ester urethane) scaffolds	ε-caprolactone, glycerol, glycolide, hexamethylene diisocyanate trimer (HDIt)	Reactive liquid molding of HDIt with the polyester triol and iron catalyst	No modification	Cutaneous wounds	Scaffolds	In vitro In vivo	N/A	[18]
Adolph et al., 2014	PUR scaffold	Lysine triisocyanate (LTI) and a polyester triol (60% caprolactone, 30% glycolide, and 10% lactide)	Reactive liquid molding of the crosslinker and calcium stearate pore opener	Plasma treatment in the presence of carboxymethylcellulose (CME)	Cutaneous wounds	Scaffolds	In vitro In vivo	Square: 6.25 cm^2^	[19]
Eskandarinia et al., 2020	Electrospun polyurethane-hyaluronic (PU-HA) acid nanofiber	Polyurethane (Tecoflex EG-80A)	Electrospinning	Enriched with ethanolic extract of propolis (EEP)	Not specific	Nanofibers	In vitro In vivo	Circle: 1.1 cm diameter	[20]
Li et al., 2019	Biodegradable electroactive polyurethane–urea elastomers	Polycaprolactone (PCL2000), polyethylene glycol (PEG2050), amine-capped (AT), 1,6-Hexanediamine (HDA), hexamethylene diisocyanate (HDI)	Two-step polymerization with stannous octoate	No modification	Skin repair	Film	In vitro In vivo	Cicle: 0.7 cm diameter	[21]
Hosseinabadi et al., 2020	PU membranes films	Castor oil and CAPA polyol (CAPA 7201—Perstorp, 9051-88-1) or DEG, hexamethylene diisocyanate (HDI)	Two-step polymerization	Chain extender: diethylene glycol (DEG)	Not specific	Film	In vitro In vivo	Square: 0.64 cm^2^	[22]
Hao et al., 2016	Waterborne biodegradable polyurethane	PEG (Mn = 1450) and PCL (Mn = 2000), LDI, PDO and L-lysine	Two-step polymerization	Chain extender: L-lysine	Not specific	Waterborne	In vitro	N/A	[23]
Liu et al., 2017	Porous polyurethane-urea foam (PUUF)	PEG, HMDI, 4, 4′-diaminodicyclohexylmethane (PACM)	Polymerization with stannous octanoate and soaking	Urea formation	Skin damage	Foam	In vitro In vivo	Square: 1 cm^2^	[24]
Guo et al., 2012	Electrospun PVDF/PU scaffold	PVDF powder and PU grains	Electrospinning	Piezoelectric PU	Skin wounds	Scaffolds	In vitro In vivo	N/A	[25]
Chen et al., 2017	Electrospun polyurethane fiber mats	Polyurethane (Mw = 8000)	Electrospinning	Mupirocin incorportaion	Burn wounds	Scaffolds	In vitro In vivo	Insition: 0.6 cm	[26]
Li et al., 2017	Amphiphilic biodegradable block polyurethane based on PLA and PEG foam	PLA (Mw = 9 × 10^4^), poly(ethylene glycol), 1,6-hexamethylene diisocyanate (HDI)	Freeze-drying method	Alternating block PU	Skin wound	Foam	In vitro In vivo	Square: 1 cm^2^	[27]
Khandwekar & Rho, 2012	PU films	Medical-grade polyurethane (Tecoflex)	Polymerization and surface modification	Cationic, anionic, and zwitterionic surfaces	Not specific	Film	In vitro In vivo	N/A	[28]
Heit et al., 2012	PU Foam	Commercial foams (GranuFoam; Kinetic Concepts)	Manufacture procedure	Pore size	Diabetic wounds	Foam	In vivo	Square: 1 cm^2^	[29]
Adolph et al., 2016	Injected PUR scaffolds	Glycolide and D,L-lactide, lysine triisocyanate-poly(ethylene glycol) (LTI-PEG) prepolymer	Two-component reactive liquid molding of LTI–PEG prepolymer	Sucrose (40% and 70%)	Cutaneous wounds	Injectable scaffolds	In vivo	Square: 9 cm^2^	[30]
Gholami & Yeganeh, 2020	Vegetable oil-based polyurethanes	Cyclic carbonated soybean oil (CSBO), CO, IPDI	Polymerization with DBTDL	Quaternary ammonium salts (QASs)	Tissue damage by skin and soft tissue infections	Film	In vitro In vivo	Square: 1.5 cm^2^	[31]

**Table 2:** Mechanical and physicochemical properties of different polyurethanes for wound dressing applications.

Reference	Dressing Type	Elongation at Break (%)	Tensile Strength (MPa)	Water Absorption (%)	Contact Angle (°)	Water Vapor Transmission Rate (g/m^2^·Day)
[16]	Film	-	-	-	-	PU membrane: 50.2 PU25/SC75: 4025.8 PU25/SC55: 3282.0 PU25/SC45: 2028.3 PU40/SC40: 954.8
[21]	Film	-	-	Ranged from 58 to 106	PCL-PEG-AT0: 25° PCL-PEG-AT6: 52° PCL-PEG-AT12: 66° PCL-PEG-AT18: 81°	-
[22]	Film	CAPA-based PU: about 550 Castor oil-based PU: about 100	CAPA-based PU: about 4 ± 0.3 Castor oil-based PU: about 1.7 ± 0.01	CAPA-based PU: 5.67 Castor oil-based PU: 0.76	CAPA-based PU: 70 ± 5 Castor oil-based PU: 80 ± 5	CAPA-based PU: 260 ± 20 Castor oil-based PU: 285 ± 20
[27]	Film	PUL15-a-E60: 995.92 PUL15-a-E80: 548.01 PUL22-a-E60: 827.69 PUL22-a-E80: 169.04 PUL15-r-E60: 79.74 PUL15-r-E80: 61.56 PUL22-r-E60: 895.48 PUL22-r-E80: 844.08	PUL15-a-E60: 5.56 PUL15-a-E80: 5.29 PUL22-a-E60: 6.89 PUL22-a-E80: 6.27 PUL15-r-E60: 3.18 PUL15-r-E80: 2.70 PUL22-r-E60: 6.33 PUL22-r-E80: 5.80	PUL8-a-E60: up to 600 PUL8-a-E80: up to 700 PUL15-a-E60: up to 500 PUL15-a-E80: up to 700 PUL22-a-E60: up to 600 PUL22-a-E80: up to 600 PUL8-r-E60: up to 500 PUL8-r-E80: up to 600 PUL15-r-E60: up to 450 PUL15-r-E80: up to 500 PUL22-r-E60: up to 500 PUL22-r-E80: up to 500	PUL8-a-E60: 44.1 ± 1.0 PUL8-a-E80: 41.4 ± 0.5 PUL15-a-E60: 52.9 ± 1.3 PUL15-a-E80: 48.9 ± 0.7 PUL22-a-E60: 57.8 ± 1.1 PUL22-a-E80: 55.3 ± 1.3 PUL8-r-E60: 50.5 ± 0.9 PUL8-r-E80: 46.8 ± 1.3 PUL15-r-E60: 55.7 ± 1.4 PUL15-r-E80: 49.7 ± 1.8 PUL22-r-E60: 61.8 ± 0.8 PUL22-r-E80: 60.4 ± 2.0	-
[28]	Film	-	-		Base: 91.4 ± 2.2 Cationic: 64 ± 2.0 Zwitterionic: 29 ± 2.5 Anionic: 48.4 ± 2.7	
[31]	Film	PUWD2 (dry): 330.0 ± 7.1 PUWD2(wet): 394.2 ± 5.3 PUWD3 (dry): 260.4 ± 7.2 PUWD3 (wet): 350.1 ± 12.2 PUWD4 (dry): 142 ± 5.2 PUWD4 (wet): 149.3 ± 7.8	PUWD2 (dry): 17.32 ± 0.61 PUWD2(wet): 5.41 ± 0.31 PUWD3 (dry): 14.37 ± 0.21 PUWD3 (wet): 2.89 ± 0.32 PUWD4 (dry): 0.11 ± 0.02 PUWD4 (wet): 0.11 ± 0.03	PUWD2: 49 ± 1.1 PUWD3: 18 ± 0.8 PUWD4: 2.1 ± 0.2	PUWD2: 37 ± 5 PUWD3: 49 ± 4 PUWD4: 85 ± 3	PUWD2: 390 ± 9 PUWD3: 191 ± 8 PUWD4: 39 ± 5
[15]	Foam	PU: 91 ± 3.5 PU-NL: 96 ± 5.6 PU-NL/EEP: 73 ± 3.9	PU: 0.75 ± 0.08 PU-NL: 0.91 ± 0.1 PU-NL/EEP: 0.82 ± 0.09	PU-NL: 267 PU-NL/EEP: 242	PU: 98.3 ± 5.8° PU-NL: 51.1 ± 4.9° PU-NL/EEP: 50.1 ± 2.1°	-
[17]	Foam	PU-control: 372 ± 12 PU/10WEP: 377 ± 14 PU/20WEP:384 ± 29 PU/30WEP: 434 ± 22	PU-control:5.26 ± 0.40 PU/10WEP: 4.79 ± 0.21 PU/20WEP: 2.91 ± 0.47 PU/30WEP: 2.99 ± 0.1	PU-control: 243 PU/10WEP:229 PU/20WEP:218 PU/30WEP: 207	PU-control: 114.52 ± 2.31 PU/10WEP: 52.41 ± 1.82 PU/20WEP: 48.81 ± 3.57 PU/30WEP: 35.53 ± 1.65	-
[24]	Foam	PUUF: about 97 CaduMedi: about 143	PUUF: 0.246 CaduMedi: about 0.116	PUU film: 88.47 in 10 min PUUF: 1310.8 in 10 min CaduMedi: 1331.69	Rapidly spread on the surface and permeating into the wound dressing in a second time	-
[29]	Foam	Small pore size foam > medium and large pore size foam	Small pore size foam > medium and large pore size	-	-	-
[30]	Injectable scaffolds	-	-	-	-	-
[11]	Nanofibers	PU/CMC: 171.52 PU/CMC/5: 167.02 PU/CMC/10: 169.71 PU/CMC/15: 200.2 PU/CMC/20: 232.88	PU/CMC: 18.5 PU/CMC/5: 21 PU/CMC/10: 22.2 PU/CMC/15: 24.9 PU/CMC/20: 26.8	PU70/CMC30: 488.11 PU80/CMC20: 469.47 PU90/CMC10: 411.36	-	PU: 497.28 PU70/CMC30: 1716.65–1987.01 PU80/CMC20: 1600.13–2074.62
[14]	Nanofibers	-	PU-Fg: 3.4 ± 0.3 PVA-Br: 21.4 ± 0.5 Sandwich: 15.8 ± 0.2	PU-Fg: 5.3	-	-
[20]	Nanofibers	PU: 354.5 ± 15.7 PU-HA: 360.1 ± 12.2 PU-HA/0.5% EEP: 379.8 ± 23.6 PU-HA/1% EEP: 382.2 ± 14.3 PU-HA/2% EEP: 453.6 ± 38.5	PU: 5.42 ± 1.4 PU-HA: 5.05 ± 0.8 PU-HA/0.5% EEP: 4.91 ± 0.5 PU-HA/1% EEP: 4.86 ± 0.9 PU-HA/2% EEP: 3.07 ± 1.1	PU: 35.21 ± 9.5 PU-HA: 74.68 ± 11.8 PU-HA/0.5% EEP: 72.11 ± 5.1 PU-HA/1% EEP: 65.54 ± 8.0 PU-HA/ 2% EEP: 51.06 ± 4.2	PU: 118.2° ± 6.2 PU-HA:43.8° ± 5.9 PU-HA/0.5% EEP: 47.6° ± 11.5 PU-HA/1% EEP: 52.2° ± 6.8 PU-HA/2% EEP: 67.2° ± 7.2	-
[13]	Porous membrane	HTPM: 424.3 CTPM: 194.6	HTPM: 2.07 CTPM: 0.21	-	-	HTPM: 2265 g per m^2^ per day CTPM: 528 g per m^2^ per day
[12]	Scaffolds	Dry samples 0.8/1: 7 0.47/1: 8 Wet samples 0.8/1: 35 0.47/1: 38	Dry samples 0.8/1: 34 0.47/1: 26 Wet samples 0.8/1: 10 0.47/1: 7	C8P2: 118.36 ± 4.9 C7P3: 100.06 ± 5.6	C8P2 and C7P3 80° ± 10°	-
[19]	Scaffolds	-	-	-	Plasma treatment significantly decreased the contact angle from 66° to 46°	-
[25]	Scaffolds	PU: 188.71 ± 22.40 PU/PVDF (3/1): 156.09 ± 31.72 PU/PVDF (2/1): 123.78 ± 46.56 PU/PVDF (1/1): 107.94 ± 25.80 PU/PVDF (1/2): 88.40 ± 26.41 PU/PVDF (1/3): 94.75 ± 20.00 PVDF: 76.47 ± 36.46	PU: 9.632 ± 0.927 PU/PVDF (3/1): 7.433 ± 1.106 PU/PVDF (2/1): 6.860 ± 0.976 PU/PVDF (1/1): 5.984 ± 1.249 PU/PVDF (1/2): 5.562 ± 0.884 PU/PVDF (1/3): 4.107 ± 1.364 PVDF: 4.016 ± 0.732	-	-	-
[26]	Scaffolds	Pu: 455.26 Pu/2%mu: 218.16 Pu/5%mu: 223.59	Pu: 8.88 Pu/2%mu: 6.29 Pu/5%mu: 5.29	-	-	Pu: 2975.13 ± 61.76 Pu/2%mu: 2810.68 ± 88.57 Pu/5%mu: 2892.89 ± 58.63
[23]	Waterborne	LWPU17: 1608 ± 15 LWPU25: 2511 ± 24 LWPU33: 2120 ± 12 LWPU45: 2050 ± 21	LWPU17: 17.8 ± 1.2 LWPU25: 12.3 ± 1.5 LWPU33: 16.8 ± 0.7 LWPU45: 15.6 ± 1.6	-	LWPUs: 72°–90°	-

**Table 3:** Analysis of tests for the evaluation of the performance of polyurethanes at each stage of the wound-healing process.

Reference	Animal Model	Injury Model	Dressing Change	Time (Days)	Techniques Performed	Hemostasis	Inflammation	Proliferation	Remodeling	Wound Closure (%)	Conclusions
[11]	Wistar rats	Full-thickness wound model	Not specific	14	Histological analysis	NO	YES	YES	YES	Gauze bandage: 32.1 ± 0.2 PU/CMC: 51.4 ± 0.4 PU/CMC/5: 71 ± 0.14 PU/CMC/10: 87.64 ± 1.02 PU/CMC/15: 95.05 ± 0.24 PU/CMC/20: 95.11 ± 0.2%	A good dual anti-inflammatory–antimicrobial wound dressing candidate for improving diabetic wound-healing
[12]	Wistar rat	Full-thickness wound model	Not specific	21	Hemostasis: in vitro. Histological analysis	YES	YES	YES	YES	Control group: 82 ± 3.91% C7P3: 100 ± 4.12%	C7P3 was observed to be biocompatible on sub-cutaneous implantation, which was supported by scaffold integration with tissue and presence of blood vessels
[13]	BALB/c mice	Full-thickness wound model	Every other day	7	Angiogenesis and proliferation: western blot; granulation thickness: histological analysis	NO	NO	YES	YES	Control: 60.3% Vaseline gauze: 72.4% CTPM: 79.4% HTPM: 91.9%	The membranes favored granulation tissue formation, wound re-epithelialization, and angiogenesis
[14]	Sprague–Dawley (SD) rats	Ischemia–reperfusion (I/R) injury	Every day	10	Histological analysis	NO	YES	NO	YES	No data	The dressing decreased bleeding, inflammation, and tissue infiltration in the dermis area and epidermis induced due to bedsore
[15]	Wistar rats	Full-thickness wound model	Not specific	12	Histological analysis	NO	NO	YES	NO	Control: ~60% PU: ~68% PU-NL: ~72% PU-NL/EEP: ~90%	PU-NL/EEP-promoted better skin wound-healing
[16]	Balb/c mice	Full-thickness wound model	Not specific	7	Histological analysis, immunohistochemistry and immunofluorescence and western blot	NO	YES	YES	YES	MP: ~95.6% Blank: 34.8% EHP: 53.2% HP: 73.4% LP: 59.0% ELP: 46.0%	Application of MP-PU membranes could maintain a suitable moist environment in the wound that could enhance the wound contraction and tissue regeneration, thereby accelerating wound-healing
[17]	Wistar rats	Full-thickness wound model	Not specific	15	Histological analysis	NO	YES	YES	NO	Control: 79.03% PU foam: 91.5% PU/30WEP: 94.32%	The increase of propolis concentration caused enhancement of the antibacterial activity against *E. coli* and *S. aureus*. The propolis-coated wound dressing exhibited significant enhancement of in vivo wound-healing
[18]	Sprague–Dawley (SD) rats	Cutaneous repair model	Not specific	21	Histological analysis, collagen: PCR; inflammation: modulation of macrophages	NO	YES	YES	YES	-	Scaffolds with a substrate modulus promoted increased deposition and random orientation of collagen, angiogenesis, and regenerative macrophage. Additionally, Wnt signaling was down-regulated on scaffolds
[19]	Yorkshire pigs	Full-thickness wound model	Every 2–3 days	15	Histological analysis	NO	YES	YES	YES	-	PUR scaffolds do not adversely affect the wound-healing process in porcine excisional wounds. The results suggest that all wounds were moving into the remodeling phase by day 15
[20]	Wistar rats	Full-thickness wound model	Not specific	21	Histological analysis	NO	YES	YES	YES	PU: 93.89 ± 0.2% PU-HA/1% EEP: 100%	The PU-HA/1% EEP exhibited higher antibacterial activity against S. aureus and E. coli in comparison with the PU and PU-HA dressings. Besides, the PU-HA/1% EEP sample caused considerable acceleration of Wistar rat skin excision healing
[21]	Kunming mice	Full-thickness wound model	Not specific	14	Histological analysis	NO	YES	YES	YES	Tegaderm™: ~90 PCL-PEG-AT0: ~98 PCL-PEG-AT12: 100 PCL-PEG-AT12/VCM: 100	PCL-PEG-AT12 film shows a prominent wound-healing effect
[22]	Wistar rats	Full-thickness wound model	Every second day	13	Histological analysis	NO	YES	NO	YES	Gauze: 68% Castor oil Pus: 99% CAPA-based Pus: 99%	This dressing can be used as the secondary dressing or applied to simple wounds with small amounts of exudates
[23]	-	-	-	-	In vitro evaluation	YES	YES	YES	NO	-	The LWPU films showed suitable mechanical properties, low cytotoxicity, good hemocompatibility and cytocompatibility. LWPUs elicited a transition of macrophages from a pro-inflammation to a wound-healing phenotype
[24]	Sprague–Dawley (SD) rats	Full-thickness wound model	Every second day	13	Histological analysis	YES	YES	YES	YES	Gauze: 35.44% CaduMedi: 98% PUUF: 98%	The results showed that PUUF can accelerate hemostasis and adsorb abundant wound exudates to build a regional moist environment beneficial for wound-healing
[25]	Sprague–Dawley (SD) rats	Subcutaneous implantation	Not specific	14	Histological analysis	NO	NO	YES	NO	-	The nonpiezoelectric-excited PU/PVDF scaffolds and the piezoelectric-excited PU scaffolds showed no significant differences in fibroblast activities
[26]	Sprague–Dawley rats	Full-thickness wound model	No	3	Histological analysis, cytokine expression: PCR	YES	YES	YES	YES	-	Increasing the content of mupirocin, the average of diameter did not show much change. There appears to be no obvious differences in the number of cells between PU and mixed PU/mupirocin scaffolds
[27]	Sprague–Dawley rats	Full-thickness wound model	Every second day	14	Histological analysis	NO	YES	YES	NO	Gauze: ~55% PULA-alt-PEG: ~98% PULA-ran-PEG: ~80%	The higher water absorption with gel formation of the alternating block polyurethanes would be good for wound-healing. It ensures that the dressings will not adhere to the wound tissue
[28]	Sprague–Dawley rats	Subcutaneous implantation-Rat cage implant system	Not specific	21	Cytokine: gene expression: PCR	YES	YES	NO	NO	-	The cationic surfaces promoted the highest rate of macrophage fusion. Anionic and zwitterionic surfaces could suppress the early macrophage response to fusogenic surface stimulus. Identify apoptosis of polyurethane adherent monocytes/macrophages as a mechanism for the removal of these cells without generating a prolonged inflammatory response
[29]	Homozygous genetically diabetic Lep/r-db/db mice (strain C57BL/KsJ-Leprdb)	Full-thickness wound model	On days 2 and 4	7	Histological analysis	NO	YES	YES	YES	No data	Larger pore sizes result in greater wound deformation, granulation tissue thickness, and induction of contractile myofibroblasts. Angiogenesis seems to be largely independent of pore size, the polyurethane foam itself induces angiogenesis
[30]	Yorkshire pigs	Full-thickness wound model	Every 2–3 days	30	Histological analysis, immunohistochemistry	NO	YES	YES	YES	I40: ~90% P40: ~85% NT: ~92%	Injected PUR scaffolds facilitate wound-healing, support tissue infiltration and matrix production, delay or reduce wound contraction, and reduce scarring in a clinically relevant animal model
[31]	Wistar rats	Full-thickness wound model	After 7 days	21	-	NO	YES	NO	NO	Gauze: ~64% PUWD4: ~71% PUWD2: ~88%	PUWD2 is probably not suitable for a bandage of heavily exudative wounds due to possibility of accumulation of exudates and consequent maceration of surrounding skin tissue

## Data Availability

The data presented in this study are available on request from the corresponding author.
